# Predictors of COVID-19 booster vaccine hesitancy among fully vaccinated adults in Korea: a nationwide cross-sectional survey

**DOI:** 10.4178/epih.e2022061

**Published:** 2022-07-22

**Authors:** Yunha Noh, Ju Hwan Kim, Dongwon Yoon, Young June Choe, Seung-Ah Choe, Jaehun Jung, Sang-Won Lee, Ju-Young Shin

**Affiliations:** 1 School of Pharmacy, Sungkyunkwan University, Suwon, Korea; 2 Department of Pediatrics, Korea University Anam Hospital, Seoul, Korea; 3 Department of Preventive Medicine, Korea University College of Medicine, Seoul, Korea; 4 Division of Life Sciences, Korea University, Seoul, Korea; 5 Department of Preventive Medicine, Gachon University College of Medicine, Incheon, Korea; 6 Department of Biohealth Regulatory Science, Sungkyunkwan University, Suwon, Korea; 7 Department of Clinical Research Design & Evaluation, Samsung Advanced Institute for Health Sciences & Technology (SAIHST), Sungkyunkwan University, Seoul, Korea

**Keywords:** Coronavirus, Vaccine hesitancy, Immunization, Public health

## Abstract

**OBJECTIVES:**

This study explored predictors of coronavirus disease 2019 (COVID-19) booster hesitancy among fully vaccinated young adults and parental COVID-19 vaccine hesitancy for their children.

**METHODS:**

This cross-sectional study administered an online survey from December 2 to December 20, 2021. We enrolled participants aged 18-49 years, for whom ≥2 weeks had passed after their initial COVID-19 vaccination. We estimated odds ratios (ORs) with 95% confidence intervals (CIs) using multivariate logistic regression to evaluate factors associated with booster/vaccine hesitancy.

**RESULTS:**

Among the 2,993 participants, 48.8% showed hesitancy (wait and see: 40.2%; definitely not: 8.7%). Booster hesitancy was more common among women (OR, 1.25; 95% CI, 1.05 to 1.50), younger people (OR, 1.44; 95% CI, 1.17 to 1.77), those with a lower education level (OR, 2.05; 95% CI, 1.10 to 3.82), those who received the mRNA-1273 vaccine type (OR, 2.01; 95% CI, 1.65 to 2.45), and those who experienced serious adverse events following previous COVID-19 vaccination (OR, 2.03; 95% CI, 1.47 to 2.80). The main reasons for booster hesitancy were concerns about safety (54.1%) and doubts about efficacy (29.8%). Among the 1,020 respondents with children aged <18 years, 65.8% were hesitant to vaccinate their children against COVID-19; hesitancy was associated with younger parental age, education level, the type of vaccine the parent received, and a history of COVID-19 infection.

**CONCLUSIONS:**

Concerns about the efficacy and safety of COVID-19 vaccines were the major barrier to booster acceptance. The initial COVID-19 vaccine type (mRNA-1273), young age, gender (women), a low education level, and adverse events after the first COVID-19 vaccine were key predictors of booster hesitancy.

## INTRODUCTION

Following the first emergency use authorization in December 2020, the coronavirus disease 2019 (COVID-19) vaccine rollout has been the top priority from a global health perspective, with more than 60% of the world population receiving at least 1 dose of a COVID-19 vaccine [1]. Despite these efforts, the effectiveness of licensed COVID-19 vaccines has been challenged following the emergence of more transmissible variants (such as Delta [B.1.617.2] and Omicron [B.1.1.529]) and the waning immunity of vaccines over time [2-4]. In response, national health authorities, including in Korea, have rolled out booster vaccines for individuals who completed the first round of COVID-19 vaccinations.

The success of the COVID-19 vaccination campaign is primarily dependent on the willingness of individuals to get vaccinated; however, many people have shown vaccine hesitancy, which is characterized by individuals’ uncertainty or refusal to get vaccinated [5]. Notably, recent data indicate a negative attitude toward the booster dose even among individuals who initially completed the first round of COVID-19 vaccinations [6]. Young adults may be more likely to spread COVID-19 since they are socially active, have low adherence to public health guidelines, and are more vaccine-hesitant than the older generation [7-9]. Due to the growing negative public perception of the recently approved booster dose, there is an urgent need to assess individuals’ hesitancy regarding booster vaccination to implement a targeted intervention for maintaining high vaccine acceptance among the younger generation.

Therefore, we explored demographic, clinical, and COVID-19-related factors associated with booster hesitancy among young adults who had completed the first round of COVID-19 vaccinations in Korea. In addition, we assessed parental factors related to hesitancy concerning vaccination of their children against COVID-19.

## MATERIALS AND METHODS

We conducted a cross-sectional study using individual-level data from a Gallup Panel online survey that took place from December 2 to December 20, 2021. We enrolled participants aged 18-49 years who had completed the first round of COVID-19 vaccinations approved in Korea at least 2 weeks earlier (i.e., those who received 2 doses of the BNT162b2 [Pfizer/BioNTech], mRNA-1273 [Moderna], or ChAdOx1 [AstraZeneca] vaccines or a single dose of the Ad26.COV2.S [J&J/Janssen] vaccine). Gallup Korea, an affiliation of Gallup International, distributed the survey via email to panels stratified by age, gender, and region (nationally representative) using the proportional allocation method. Of the 22,790 invitees, 27.9% attempted the survey (clicked the survey link) and 67.9% completed it (see Supplementary Material 1 for details). After the survey, we weighted the respondents by age, gender, and region to represent the Korean population. The total number of participants in the weighted sample was equal to that in the unweighted sample. The study was conducted according to the Strengthening the Reporting of Observational Studies in Epidemiology (STROBE) guidelines for cross-sectional studies [10].

**Figure 1.**
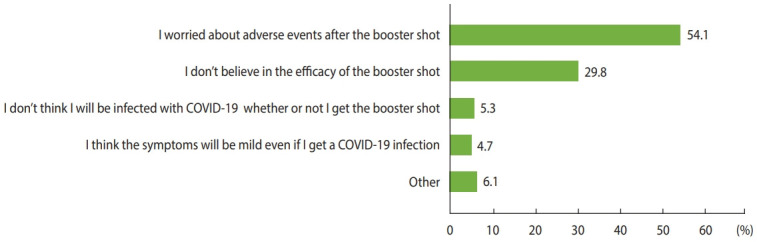
Reasons for coronavirus disease 2019 (COVID-19) booster vaccination hesitancy (n=1,462).

Our primary outcomes were the prevalence and predictors of COVID-19 booster hesitancy. We asked all participants, “How likely are you to receive the booster shot for COVID-19 once the booster vaccine is available to you?” with response options including “already received,” “as soon as possible,” “wait and see,” and “definitely not.” Vaccine hesitancy was considered to be present when respondents answered “wait and see” or “definitely not,” and acceptance was indicated by the answers “already received” and “as soon as possible.” We evaluated the following demographic and clinical factors as potential predictors of booster hesitancy: gender, age, region, education level, employment, type of COVID-19 vaccine received, history of any or serious patient-reported adverse events following COVID-19 vaccination, history of a severe allergic reaction, anticoagulant use in the past 6 months, history of COVID-19 infection, history of other vaccinations in the past 3 years, smoking status, body mass index, alcohol consumption, and comorbidities. The secondary outcome was the reason for booster hesitancy. Vaccine-hesitant participants were asked, “What is the most important reason you would not receive the booster shot?” and selected 1 main reason from a list of 5 possible answers (Figure 1). In addition, we examined parental characteristics associated with COVID-19 vaccine hesitancy for their children. All participants were asked if they had a child aged < 18 years, and if they had a child, they were asked, “How likely are you to bring your child to receive the COVID-19 vaccine?” with the same 4 response options mentioned above.

The characteristics of vaccine acceptance and hesitancy groups were reported using frequencies (percentage) for categorical variables and means (standard deviation [SD]) for continuous variables. Using univariate and multivariate logistic regression models, we estimated weighted odds ratios (ORs) with 95% confidence intervals (CIs) to evaluate the factors associated with booster/vaccine hesitancy compared to acceptance. Furthermore, 95% CIs that did not overlap 1.0 were considered to indicate statistical significance. All statistical analyses were performed using SAS version 9.4 (SAS Institute Inc., Cary, NC, USA) and Microsoft Excel (Microsoft Corp., Redmond, WA, USA).

### Ethics statement

This study was approved by the Institutional Review Board of Sungkyunkwan University (SKKU 2021-11-019), and all respondents consented to participate in the survey.

## RESULTS

Of the 2,993 participants (mean± SD, 34.6± 8.9 years; 51.6% men), 51.2% showed booster acceptance, whereas 48.8% indicated vaccine hesitancy (wait and see: 40.2%; definitely not: 8.7%) (Table 1). The most commonly received vaccine was the BNT162b2 (65.4%) vaccine, followed by the mRNA-1273 (20.7%), ChAdOx1 (8.7%), and Ad26.COV2.S (4.8%) vaccines.

The likelihood of COVID-19 booster hesitancy was higher among women (OR, 1.25; 95% CI, 1.05 to 1.50), younger respondents (OR, 1.44; 95% CI, 1.17 to 1.77 among those aged 18-29 years and OR, 1.59; 95% CI, 1.30 to 1.94 among those aged 30-39 years compared to those aged 40-49 years), those with a lower education level (OR, 2.05; 95% CI, 1.10 to 3.82 for those without high school degrees compared to those with graduate degrees), those who received the mRNA-1273 vaccine (OR, 2.01; 95% CI, 1.65 to 2.45), and those with a history of patient-reported serious adverse events after COVID-19 vaccination (OR, 2.03; 95% CI, 1.47 to 2.80), whereas the odds of hesitancy were lower for those who received the ChAdOx1 (OR, 0.32; 95% CI, 0.23 to 0.45) or Ad26.COV2.S (OR, 0.22; 95% CI, 0.14 to 0.36) vaccines, those who had received other vaccines in the past 3 years (OR, 0.70; 95% CI, 0.60 to 0.81), and patients with diabetes (OR, 0.51; 95% CI, 0.29 to 0.92) (Table 2). The main reasons for booster hesitancy were concerns about adverse events after the booster shot (54.1%), followed by doubt about the efficacy of the booster shot (29.8%) (Figure 1). The factors associated with booster refusal, which was indicated by a response of “definitely not,” were largely consistent with those associated with booster hesitancy (Supplementary Material 2).

Among the 1,020 respondents who answered that they had children aged < 18 years, 34.2% showed COVID-19 vaccine acceptance for their children, whereas 65.8% reported hesitancy (Table 3). The likelihood of COVID-19 vaccine hesitancy for children was higher among those in the younger age group (OR, 5.14; 95% CI, 3.54 to 7.48 among those aged 30-39 years compared to those aged 40-49 years) and lower for those with a lower education level (OR, 0.51; 95% CI, 0.28 to 0.91 for those without high school degrees compared to those with graduate degrees), for those who received an initial dose of the ChAdOx1 vaccine type (OR, 0.52; 95% CI, 0.35 to 0.77), and those with a history of COVID-19 infection (OR, 0.20; 95% CI, 0.06 to 0.68).

## DISCUSSION

This study explored the prevalence of COVID-19 booster hesitancy and its associated factors among those who had completed the first round of COVID-19 vaccinations when pre-registration for booster vaccination for young adults began in Korea. We found that nearly half of the survey respondents showed vaccine hesitancy and expressed concerns about adverse events and the efficacy of the booster vaccine. Our findings add to the existing data, showing that gender (women), age (those who are younger), education level (lower), the initial type of COVID-19 vaccine received (mRNA-1273), and the experience of adverse events following COVID-19 vaccination (yes) were key predictors of booster hesitancy. Moreover, some of these predictors were also identified as parental factors associated with COVID-19 vaccine hesitancy related to the respondents’ children, suggesting that targeting this hesitant group would synergistically lead to improvements in booster vaccination and COVID-19 vaccination campaigns aimed at increasing the child vaccination rate.

Few studies to date have explored the prevalence of hesitancy against the COVID-19 booster. A study in the United Kingdom reported that 8% of fully vaccinated adults were unwilling to receive a booster shot, and a similar finding was observed in Denmark [6,11]. Our finding on the unwillingness rate (indicated by a response of “definitely not”) was 8.7%, which was comparable to those reported from other regions. Although the definition of hesitancy varied by study, in this study, we considered those in the “wait and see” group to have booster hesitancy. This group is likely to include individuals with some hesitancy or a passive attitude toward receiving the booster. From a public health perspective, they are regarded as important targets for outreach or messaging since they are relatively easier to convert from hesitancy to acceptance than those in the “definitely not” group [12]. Nonetheless, the booster hesitancy rate in our study may have been overestimated since the majority of the respondents answered “wait and see.”

Moreover, hesitancy rates can fluctuate across different time points and epidemic waves. For example, surveys of United States healthcare workers showed an increase in the intention to receive the COVID-19 vaccine from 36% in November 2020 to 85% in March 2021 once vaccination began [13,14]. Conversely, the intention to receive a COVID-19 vaccine among survey respondents in Hong Kong showed a slight decrease from 44.2% to 34.8% during the first and third local epidemic waves [15]. In our study, the survey was conducted when vaccine breakthrough infections rapidly increased and Omicron variants emerged [16]. Although booster efficacy against the Omicron variant has been recently proven [17], at the time of the survey, the relatively uncertain efficacy of existing vaccines for other COVID-19 variants and breakthrough infections may have influenced a significant portion of the respondents to answer “wait and see” to discern the seriousness of the impending epidemic wave before deciding to receive a booster vaccine. In addition, the survey was conducted at the beginning of the booster vaccination campaign for young adults, which may have led to high hesitancy. Nevertheless, given that severe acute respiratory syndrome coronavirus 2 (SARS-CoV-2) variants have been predicted to occur repeatedly and that booster vaccination programs may change depending on the emergence of new variants, the results of our study are meaningful since they provide data that can be used to devise more effective booster campaigns.

Our findings on the predictors of booster hesitancy are generally in line with the findings of previous studies. Expectedly, we found that younger respondents, women, and those with a low education level, which are commonly cited as key factors for vaccine hesitancy in general [18], continued to be more likely to express COVID-19 vaccine hesitancy [19]. Recently published data have also indicated an unwillingness to accept a booster dose among women and young individuals in Czechia [20], university students in Germany [21], and those who experienced adverse events following COVID-19 vaccination in Poland [22]. Moreover, the predictors of booster hesitancy generally corresponded to those identified during the initial COVID-19 vaccine rollout. In a COVID-19 social study from the United Kingdom, those with a lower education level, those with a lower household income, or individuals from ethnic minority groups expressed hesitancy toward COVID-19 vaccination [6]. Another study of healthcare workers in the United States cited fear of potential adverse events as a major concern regarding vaccination against COVID-19 [23]. Reflecting these concerns, the experience of adverse events after initial COVID-19 vaccination was a key predictor of booster hesitancy in this study.

Our findings also filled an evidence gap related to the association between initial vaccine type and booster hesitancy. One study in Denmark found a decreased likelihood of willingness to receive a booster dose among those initially vaccinated with the mRNA-1273 vaccine, with the ChAdOx1 vaccine alone or heterologous inoculation as the reference, though the difference was not statistically significant [11]. In our study, using the BNT162b2 vaccine as a reference, we confirmed that initial vaccination with the mRNA-1273 vaccine was another key predictor of booster hesitancy. We speculate that this is potentially due to the differing risk of adverse events according to vaccine type, as suggested by a cohort study in the United States in which an increased likelihood of adverse events was reported following vaccination with the mRNA-1273 vaccine (OR, 2.00; 95% CI, 1.86 to 2.15) compared to the BNT162b2 vaccine [24]. Conversely, we found initial vaccination with non-mRNA COVID-19 vaccines to be inversely associated with booster hesitancy. This can be partly attributed to the relatively lower efficacy of the ChAdOx1 and Ad26.COV2.S vaccines than mRNA vaccines based on phase III trials [25,26]. While a formal comparison of trials has yet to be made, the general public may have perceived the former vaccines to have less of a protective effect against COVID-19. Taken together, COVID-19 booster hesitancy may rely on known differences in safety and effectiveness according to the vaccine type, indicating that the accurate communication of information about the safety and effectiveness of each vaccine may be important for encouraging vaccination. One way to overcome safety-related hesitancy could be to accept heterologous COVID-19 booster vaccination [27,28]. Lastly, individuals with self-reported diabetes and past vaccinations were less likely to be booster-hesitant. These findings can be attributed to a “healthy adherer effect” in individuals who are more likely to be adherent to therapy or who exhibit health-seeking behaviors [29].

As an exploratory analysis, this study assessed the predictors of parents’ intentions to vaccinate their children and found that some of the key predictors of booster hesitancy no longer held for vaccination against COVID-19 for their children. Notably, parents with lower education levels tended to be less hesitant when vaccinating their children, whereas lower education levels have generally been regarded as a barrier to childhood vaccination [30]. Future studies are needed to determine whether this relationship between education level and vaccine hesitancy is specific to the booster dose or applicable to other vaccines. Nevertheless, we consistently observed that young age was one of the significant predictors of parental COVID-19 vaccine hesitancy, whereas parents who received the ChAdOx1 vaccine were less likely to be hesitant toward vaccinating their children against COVID-19. Although direct comparison with our study is difficult, in a previous study investigating the predictors of caregivers’ intentions to vaccinate their children against COVID-19, a greater willingness to vaccinate was reported when the children were older, the caregiver was older, when the father completed the survey, and when parents and children had been vaccinated in the past [31].

The strengths of this study include its identification of several key predictors for barriers and facilitators related to COVID-19 booster vaccination, as well as the sample’s representativeness of the Korean adult population. In addition, this study has important public health implications since it identified the predictors of COVID-19 booster hesitancy among young adults, as they have an increased likelihood of COVID-19 transmission due to high vaccine hesitancy and active socialization. Encouraging vaccination among young adults is necessary since it increases the possibility of herd immunity and protects others from infection, especially those who are vulnerable or cannot get vaccinated. Our findings will also be important over time since several COVID-19 vaccines will be approved for booster use in the future. However, several limitations should be noted when interpreting this study’s findings. First, given the nature of the questionnaire-based survey design, this study is subject to social desirability bias, potentially leading to underestimating the true booster hesitancy rate. Indeed, this would substantially impact the estimated prevalence of hesitancy if the survey was administered in person; however, since the survey was administered via email, we believe that the impact was minimal. Second, the predictors of hesitancy identified in our study are sensitive to the point in time when the assessment took place. At the time of our survey, 3 months had not yet passed after receiving the second dose for many of the respondents, and this may have influenced many to express a passive attitude toward receiving the booster. Therefore, future studies are needed to compare hesitancy among “unboosted” individuals with that among “boosted” individuals to confirm our study’s findings. Lastly, our findings may not be generalizable to those who live outside of Korea due to differences in COVID-19 vaccination access and compliance with government policies.

In conclusion, the prevalence of booster hesitancy was found to be growing among fully vaccinated young adults in Korea. Concerns about the efficacy and safety of COVID-19 vaccines were the major barriers to booster acceptance, and those who were younger, women, had a lower education level, and had a history of adverse events following COVID-19 vaccination showed higher booster hesitancy. Furthermore, the initial COVID-19 vaccine type administered to an individual may have played a key role, as supported by the differences according to the initial vaccine type reported in our study.

## Figures and Tables

**Table 1.** Characteristics of participants

Characteristics	Total (n=2,993)	Acceptance (n=1,531)	Hesitancy (n=1,462)
Gender			
Men	1,544 (51.6)	873 (57.0)	671 (45.9)
Women	1,449 (48.4)	658 (43.0)	791 (54.1)
Age, mean±SD (yr)	34.6±8.9	35.9±8.8	33.3±8.8
Age (yr)			
18-29	1,017 (34.0)	434 (28.3)	583 (39.9)
30-39	895 (29.9)	459 (30.0)	436 (29.8)
40-49	1,081 (36.1)	638 (41.7)	443 (30.3)
Region			
Metropolitan	597 (19.9)	297 (19.4)	300 (20.5)
Urban	1,588 (53.1)	819 (53.5)	769 (52.6)
Rural	808 (27.0)	415 (27.1)	393 (26.9)
Education level			
No high school degree	59 (2.0)	20 (1.3)	39 (2.7)
High school graduate	586 (19.6)	277 (18.1)	309 (21.1)
Undergraduate degree	2,026 (67.7)	1,048 (68.5)	978 (66.9)
Graduate degree	322 (10.8)	186 (12.1)	136 (9.3)
Employment			
Employed	2,120 (70.8)	1,132 (73.9)	988 (67.6)
Housemaker/Unemployed	873 (29.2)	399 (26.1)	474 (32.4)
Type of COVID-19 vaccine			
BNT162b2	1,956 (65.4)	983 (64.2)	973 (66.6)
mRNA-1273	619 (20.7)	212 (13.8)	407 (27.8)
ChAdOx1^1^	259 (8.7)	204 (13.3)	55 (3.8)
Ad26.COV2.S	144 (4.8)	121 (7.9)	23 (1.6)
Others^2^	15 (0.5)	11 (0.7)	4 (0.3)
History of any AEs after COVID-19 vaccine	2,848 (95.2)	1,448 (94.6)	1,400 (95.8)
History of serious AEs after COVID-19 vaccine	197 (6.6)	71 (4.6)	126 (8.6)
History of severe allergic reaction	89 (3.0)	48 (3.1)	41 (2.8)
Anticoagulant use in past 6 mo	34 (1.1)	24 (1.6)	10 (0.7)
History of COVID-19 infection	26 (0.9)	17 (1.1)	9 (0.6)
History of other vaccination in the past 3 yr	1,575 (52.6)	882 (57.6)	693 (47.4)
Smoking status			
Never smoker	1,996 (66.7)	965 (63.0)	1,031 (70.5)
Ex-smoker	398 (13.3)	235 (15.3)	163 (11.1)
Current smoker	599 (20.0)	331 (21.6)	268 (18.3)
Body mass index (kg/m^2^)			
Underweight (<18.5)	195 (6.5)	82 (5.4)	113 (7.7)
Healthy weight (18.5 to <23.0)	1,289 (43.1)	597 (39.0)	692 (47.3)
Overweight (23.0 to <25.0)	598 (20.0)	325 (21.2)	273 (18.7)
Obesity (≥25.0)	911 (30.4)	527 (34.4)	384 (26.3)
Alcohol consumption (times/wk)			
≤1	2,512 (83.9)	1,255 (82.0)	1,257 (86.0)
>1	481 (16.1)	276 (18.0)	205 (14.0)
Comorbidities			
Diabetes	71 (2.4)	51 (3.3)	20 (1.4)
Hypertension	193 (6.4)	119 (7.8)	74 (5.1)
Cardiovascular diseases	14 (0.5)	9 (0.6)	5 (0.3)
Cerebrovascular diseases	17 (0.6)	10 (0.7)	7 (0.5)
Cancer	21 (0.7)	13 (0.8)	8 (0.5)
Autoimmune diseases	39 (1.3)	22 (1.4)	17 (1.2)
Dermatologic diseases	136 (4.5)	71 (4.6)	65 (4.4)
Respiratory diseases	72 (2.4)	32 (2.1)	40 (2.7)
Renal diseases	14 (0.5)	9 (0.6)	5 (0.3)
Liver diseases	39 (1.3)	27 (1.8)	12 (0.8)
Neurological diseases	2 (0.1)	2 (0.1)	0 (0.0)
Psychiatric or mental diseases	92 (3.1)	50 (3.3)	42 (2.9)
Other diseases	105 (3.5)	56 (3.7)	49 (3.4)

Values are presented as number (%). SD, standard deviation; COVID-19, coronavirus disease 2019; AE, adverse event.

1 All respondents who received a first dose of the ChAdOx1 vaccine were followed by a second dose of an mRNA vaccine (BNT162b2, n=253; mRNA-1273, n=6).

2 Cross-inoculation with mRNA vaccines (BNT162b2–mRNA-1273, n=10; mRNA-1273–BNT162b2, n=5).

**Table 2.** Characteristics associated with COVID-19 booster vaccination hesitancy (n=2,993)

Characteristics	Unadjusted	Adjusted^1^
Gender		
Men	1.00 (reference)	1.00 (reference)
Women	1.57 (1.36, 1.81)	1.25 (1.05, 1.50)
Age (yr)		
18-29	1.94 (1.63, 2.31)	1.44 (1.17, 1.77)
30-39	1.37 (1.15, 1.64)	1.59 (1.30, 1.94)
40-49	1.00 (reference)	1.00 (reference)
Region		
Metropolitan	1.00 (reference)	1.00 (reference)
Urban	0.93 (0.77, 1.12)	0.92 (0.76, 1.13)
Rural	0.94 (0.76, 1.16)	0.85 (0.68, 1.07)
Education level		
No high school degree	2.68 (1.50, 4.81)	2.05 (1.10, 3.82)
High school graduate	1.53 (1.16, 2.01)	1.15 (0.84, 1.57)
Undergraduate degree	1.28 (1.01, 1.62)	1.11 (0.85, 1.43)
Graduate degree	1.00 (reference)	1.00 (reference)
Employment		
Employed	1.00 (reference)	1.00 (reference)
Housemaker/Unemployed	1.36 (1.16, 1.59)	1.07 (0.88, 1.30)
Type of COVID-19 vaccine		
BNT162b2	1.00 (reference)	1.00 (reference)
mRNA-1273	1.94 (1.60, 2.34)	2.01 (1.65, 2.45)
ChAdOx1^2^	0.27 (0.20, 0.37)	0.32 (0.23, 0.45)
Ad26.COV2.S	0.19 (0.12, 0.30)	0.22 (0.14, 0.36)
Others^3^	0.37 (0.12, 1.17)	0.32 (0.10, 1.06)
History of AEs after COVID-19 vaccination	1.30 (0.93, 1.82)	1.11 (0.77, 1.59)
History of serious AEs after COVID-19 vaccination	1.94 (1.44, 2.62)	2.03 (1.47, 2.80)
History of severe allergic reaction	0.89 (0.59, 1.36)	1.04 (0.66, 1.64)
Anticoagulant use in past 6 mo	0.43 (0.20, 0.90)	0.52 (0.22, 1.24)
History of COVID-19 infection	0.55 (0.25, 1.25)	0.65 (0.27, 1.52)
History of other vaccination in the past 3 yr	0.66 (0.57, 0.77)	0.70 (0.60, 0.81)
Smoking status		
Never smoker	1.00 (reference)	1.00 (reference)
Ex-smoker	0.65 (0.52, 0.81)	0.93 (0.73, 1.20)
Current smoker	0.76 (0.63, 0.91)	0.99 (0.80, 1.24)
Body mass index (kg/m^2^)		
Underweight (<18.5)	1.00 (reference)	1.00 (reference)
Healthy weight (18.5 to <23.0)	0.84 (0.62, 1.14)	0.95 (0.69, 1.31)
Overweight (23.0 to <25.0)	0.61 (0.44, 0.84)	0.82 (0.58, 1.17)
Obesity (≥25.0)	0.53 (0.39, 0.72)	0.74 (0.52, 1.04)
Alcohol consumption (times/wk)		
≤1	1.00 (reference)	1.00 (reference)
>1	0.74 (0.61, 0.90)	0.84 (0.67, 1.05)
Comorbidities		
Diabetes	0.40 (0.24, 0.68)	0.51 (0.29, 0.92)
Hypertension	0.64 (0.47, 0.86)	1.10 (0.78, 1.56)
Cardiovascular diseases	0.57 (0.19, 1.72)	0.92 (0.26, 3.22)
Cerebrovascular diseases	0.73 (0.28, 1.93)	1.35 (0.42, 4.28)
Cancer	0.63 (0.26, 1.52)	0.50 (0.20, 1.27)
Autoimmune diseases	0.81 (0.43, 1.53)	0.89 (0.44, 1.79)
Dermatologic diseases	0.96 (0.68, 1.35)	0.81 (0.56, 1.18)
Respiratory diseases	1.32 (0.82, 2.11)	1.55 (0.92, 2.59)
Renal diseases	0.58 (0.20, 1.74)	1.03 (0.30, 3.60)
Liver diseases	0.46 (0.23, 0.92)	0.65 (0.32, 1.35)
Psychiatric or mental diseases	0.88 (0.58, 1.33)	0.80 (0.51, 1.25)
Other diseases	0.91 (0.61, 1.34)	1.05 (0.69, 1.60)

Values are presented as odds ratio (95% confidence interval). COVID-19, coronavirus disease 2019; AE, adverse event.

1 Adjusted for covariates presented in Table 2.

2 All respondents who received a first dose of the ChAdOx1 vaccine were followed by a second dose of an mRNA vaccine (BNT162b2, n=253; mRNA-1273, n=6).

3 Cross-inoculation with mRNA vaccines (BNT162b2–mRNA-1273, n=10; mRNA-1273–BNT162b2, n=5).

**Table 3.** Parental characteristics associated with hesitancy concerning vaccination of their children against COVID-19 (n=1,020)

Parental characteristics	Acceptance (n=349)	Hesitancy (n=671)	Unadjusted	Adjusted^1^
Gender				
Men	193 (55.3)	361 (53.8)	1.00 (reference)	1.00 (reference)
Women	156 (44.7)	310 (46.2)	1.06 (0.82, 1.38)	0.90 (0.64, 1.25)
Age (yr)				
18-29	10 (2.9)	15 (2.2)	1.14 (0.50, 2.56)	1.35 (0.57, 3.22)
30-39	47 (13.5)	270 (40.2)	4.35 (3.08, 6.14)	5.14 (3.54, 7.48)
40-49	292 (83.7)	386 (57.5)	1.00 (reference)	1.00 (reference)
Region				
Metropolitan	60 (17.2)	118 (17.6)	1.00 (reference)	1.00 (reference)
Urban	180 (51.6)	359 (53.5)	1.01 (0.71, 1.45)	1.05 (0.71, 1.56)
Rural	109 (31.2)	194 (28.9)	0.91 (0.61, 1.34)	0.93 (0.61,1.43)
Education level				
No high school degree	3 (0.9)	1 (0.1)	0.15 (0.02, 1.52)	0.10 (0.01, 1.20)
High school graduate	43 (12.3)	53 (7.9)	0.57 (0.34, 0.97)	0.51 (0.28, 0.91)
Undergraduate degree	255 (73.1)	513 (76.5)	0.93 (0.64, 1.35)	0.82 (0.55, 1.23)
Graduate degree	48 (13.8)	104 (15.5)	1.00 (reference)	1.00 (reference)
Employment				
Employed	287 (82.2)	533 (79.4)	1.00 (reference)	1.00 (reference)
Housemaker/Unemployed	62 (17.8)	138 (20.6)	1.20 (0.86, 1.67)	1.40 (0.93, 2.11)
Type of COVID-19 vaccine				
BNT162b2	199 (57.0)	401 (59.8)	1.00 (reference)	1.00 (reference)
mRNA-1273	63 (18.1)	141 (21.0)	1.11 (0.79, 1.56)	1.12 (0.77, 1.63)
ChAdOx1^2^	66 (18.9)	82 (12.2)	0.62 (0.43, 0.89)	0.52 (0.35, 0.77)
Ad26.COV2.S	17 (4.9)	43 (6.4)	1.26 (0.70, 2.26)	0.58 (0.30, 1.14)
Others^3^	4 (1.1)	4 (0.6)	0.50 (0.12, 2.01)	0.31 (0.07,1.49)
History of AEs after COVID-19 vaccination	332 (95.1)	657 (97.9)	2.40 (1.17, 4.93)	2.18 (0.99, 4.79)
History of serious AEs after COVID-19 vaccination	19 (5.4)	51 (7.6)	1.43 (0.83, 2.46)	1.38 (0.78, 2.46)
History of severe allergic reaction	16 (4.6)	18 (2.7)	0.57 (0.29, 1.14)	0.60 (0.28, 1.28)
History of COVID-19 infection	9 (2.6)	5 (0.7)	0.28 (0.09, 0.85)	0.20 (0.06, 0.68)
History of other vaccination in the past 3 yr	219 (62.8)	442 (65.9)	1.15 (0.88,1.50)	1.07 (0.80, 1.44)

Values are presented as number (%) or odds ratio (95% confidence interval). COVID-19, coronavirus disease 2019; AE, adverse event.

1 Adjusted for covariates presented in Table 3.

2 All respondents who received a first dose of the ChAdOx1 vaccine were followed by a second dose of an mRNA vaccine (BNT162b2, n=253; mRNA-1273, n=6).

3 Cross-inoculation with mRNA vaccines (BNT162b2–mRNA-1273, n=10; mRNA-1273–BNT162b2, n=5).

## SUPPLEMENTARY MATERIALS

Supplementary Material 1. Survey details

Supplementary Material 2 Characteristics associated with the refusal of COVID-19 booster vaccination
