# Supplementary material for: Predictors of COVID-19 booster vaccine hesitancy among fully vaccinated adults in Korea: a nationwide cross-sectional survey
Source: Epidemiol Health. 2022 Jul 22;44:e2022061. doi: 10.4178/epih.e2022061 (PMC9754905; doi:10.4178/epih.e2022061)
Supplement: Supplementary Material 2 — Characteristics associated with the refusal of COVID-19 booster vaccination [file epih-44-e2022061-suppl2.docx]

**Supplementary Material 2.** Characteristics associated with the refusal of COVID-19 booster vaccination

| **Characteristics (n=1,790)** | **Acceptance**  **(n=1,531)** | **Refusal**  **(n=259)** | **Unadjusted ORs (95% CI)** | **Adjusted ORs**^‡^ **(95% CI)** |
| --- | --- | --- | --- | --- |
| Sex, n (%) |  |  |  |  |
| Male | 873 (57.0) | 129 (49.8) | 1.00 (ref) | 1.00 (ref) |
| Female | 658 (43.0) | 130 (50.2) | 1.34 (1.03-1.74) | 1.20 (0.86-1.68) |
| Age, mean (SD), year | 35.9 (8.8) | 31.7 (8.6) | ‒ | ‒ |
| Age group, n (%), year |  |  |  |  |
| 18-29 | 434 (28.3) | 126 (48.6) | 3.32 (2.37-4.66) | **2.73 (1.83-4.08)** |
| 30-39 | 459 (30.0) | 77 (29.7) | 1.92 (1.34-2.77) | **2.66 (1.78-3.97)** |
| 40-49 | 638 (41.7) | 56 (21.6) | 1.00 (ref) | 1.00 (ref) |
| Region, n (%) |  |  |  |  |
| Metropolitan | 297 (19.4) | 56 (21.6) | 1.00 (ref) | 1.00 (ref) |
| Urban | 819 (53.5) | 141 (54.4) | 0.91 (0.65-1.28) | 0.85 (0.58-1.23) |
| Rural | 415 (27.1) | 62 (23.9) | 0.79 (0.53-1.16) | 0.65 (0.42-1.00) |
| Education level, n (%) |  |  |  |  |
| No high school degree | 20 (1.3) | 14 (5.4) | 6.56 (2.87-14.96) | **5.77 (2.26-14.77)** |
| High school graduate | 277 (18.1) | 56 (21.6) | 1.88 (1.09-3.24) | 1.15 (0.61-2.16) |
| Undergraduate degree | 1,048 (68.5) | 169 (65.3) | 1.51 (0.92-2.46) | 1.14 (0.66-1.95) |
| Graduate degree | 186 (12.1) | 20 (7.7) | 1.00 (ref) | 1.00 (ref) |
| Employment, n (%) |  |  |  |  |
| Employed | 1,132 (73.9) | 175 (67.6) | 1.00 (ref) | 1.00 (ref) |
| Housemaker/Unemployed | 399 (26.1) | 85 (32.8) | 1.38 (1.04-1.83) | 1.07 (0.75-1.53) |
| Type of COVID-19 vaccine, n (%) |  |  |  |  |
| BNT162b2 | 983 (64.2) | 161 (62.2) | 1.00 (ref) | 1.00 (ref) |
| mRNA-1273 | 212 (13.8) | 88 (34.0) | 2.54 (1.89-3.43) | **2.83 (2.04-3.94)** |
| ChAdOx1^*^ | 204 (13.3) | 7 (2.7) | 0.21 (0.10-0.45) | **0.29 (0.13-0.64)** |
| Ad26.COV2.S | 121 (7.9) | 2 (0.8) | 0.10 (0.03-0.42) | **0.12 (0.03-0.52)** |
| Others^†^ | 11 (0.7) | 1 (0.4) | 0.57 (0.08-4.36) | 0.28 (0.03-2.46) |
| History of AEs after COVID-19 vaccination, n (%) | 1,448 (94.6) | 242 (93.4) | 0.82 (0.48-1.40) | 0.66 (0.37-1.20) |
| History of serious AEs after COVID-19 vaccination, n (%) | 71 (4.6) | 39 (15.1) | 3.66 (2.41-5.54) | **4.74 (2.94-7.65)** |
| History of severe allergic reaction, n (%) | 48 (3.1) | 6 (2.3) | 0.73 (0.31-1.72) | 0.78 (0.30-2.01) |
| Anticoagulant use in past 6 months, n (%) | 24 (1.6) | 0 | ‒ | ‒ |
| History of COVID-19 infection, n (%) | 17 (1.1) | 2 (0.8) | 0.71 (0.16-3.04) | 1.03 (0.22-4.83) |
| History of other vaccination in the past 3 years, n (%) | 882 (57.6) | 120 (46.3) | 0.63 (0.49-0.83) | **0.64 (0.48-0.86)** |
| Smoking status, n (%) |  |  |  |  |
| Never smoker | 965 (63.0) | 167 (64.5) | 1.00 (ref) | 1.00 (ref) |
| Ex-smoker | 235 (15.3) | 32 (12.4) | 0.79 (0.53-1.18) | 1.55 (0.97-2.50) |
| Current smoker | 331 (21.6) | 60 (23.2) | 1.05 (0.76-1.45) | **1.64 (1.10-2.43)** |
| Body mass index, , n (%), kg/m^2^ |  |  |  |  |
| Underweight (<18.5) | 82 (5.4) | 20 (7.7) | 1.00 (ref) | 1.00 (ref) |
| Healthy weight (18.5 to <23) | 597 (39.0) | 121 (46.7) | 0.83 (0.49-1.40) | 1.03 (0.58-1.82) |
| Overweight (23 to <25) | 325 (21.2) | 64 (24.7) | 0.80 (0.46-1.40) | 1.21 (0.65-2.27) |
| Obesity (≥25) | 527 (34.4) | 55 (21.2) | 0.43 (0.24-0.75) | 0.64 (0.34-1.21) |
| Alcohol consumption, n (%) |  |  |  |  |
| ≤1 times/week | 1,255 (82.0) | 223 (86.1) | 1.00 (ref) | 1.00 (ref) |
| >1 times/week | 276 (18.0) | 36 (13.9) | 0.74 (0.51-1.07) | 0.83 (0.54-1.28) |
| Comorbidities, n (%) |  |  |  |  |
| Diabetes | 51 (3.3) | 3 (1.2) | 0.35 (0.11-1.11) | 0.53 (0.15-1.92) |
| Hypertension | 119 (7.8) | 10 (3.9) | 0.48 (0.25-0.93) | 0.78 (0.37-1.66) |
| Cardiovascular diseases | 9 (0.6) | 0 | ‒ | ‒ |
| Cerebrovascular diseases | 10 (0.7) | 2 (0.8) | 1.18 (0.26-5.42) | 2.37 (0.37-15.08) |
| Cancer | 13 (0.8) | 2 (0.8) | 0.92 (0.21-4.06) | 0.77 (0.14-4.25) |
| Autoimmune diseases | 22 (1.4) | 2 (0.8) | 0.53 (0.12-2.27) | 0.68 (0.14-3.28) |
| Dermatologic diseases | 71 (4.6) | 7 (2.7) | 0.57 (0.26-1.25) | 0.48 (0.21-1.12) |
| Respiratory diseases | 32 (2.1) | 4 (1.5) | 0.74 (0.26-2.10) | 0.97 (0.31-3.07) |
| Renal diseases | 9 (0.6) | 0 | ‒ | ‒ |
| Liver diseases | 27 (1.8) | 2 (0.8) | 0.43 (0.10-1.84) | 0.93 (0.20-4.32) |
| Neurological diseases | 2 (0.1) | 0 | ‒ | ‒ |
| Psychiatric or mental diseases | 50 (3.3) | 3 (1.2) | 0.35 (0.11-1.12) | **0.28 (0.08-0.96)** |
| Other diseases | 56 (3.7) | 5 (1.9) | 0.51 (0.20-1.30) | 0.66 (0.25-1.77) |

**Abbreviation:** AE, adverse event; CI, confidence interval; COVID-19, coronavirus disease 2019; OR, odds ratio.

*All respondents who received a first dose of ChAdOx1 vaccine were followed by a second dose of an mRNA vaccine (BNT162b2, n=253; mRNA-1273, n=6). ^†^Cross-inoculation among mRNA vaccines (BNT162b2–mRNA-1273, n= 10; mRNA-1273–BNT162b2, n=5).
